# Shortening MRI scanning time for acute ischemic stroke: analysis of the effect of 3.0T MRI compressed sensing deep learning reconstruction

**DOI:** 10.1007/s10140-026-02496-w

**Published:** 2026-06-04

**Authors:** Xinxin Jiang, Feixiang Shang, Junqi Liu, Qiuzi Qin, Pei Li, Zhentao Chen, Li Li, Weijun Qian

**Affiliations:** 1 Department of Imaging, Kaifeng Central Hospital, NO.85, Riverway Street, Shunhe Hui District, Kaifeng City, Henan Province 475000 China; 2 Image Department, Canon Medical Systems (China), Beijing, 100027 China

**Keywords:** Acute ischemic stroke, 3.0T MRI, CS-DLR, Image quality, Scanning time shortening

## Abstract

**Background:**

Acute ischemic stroke requires rapid and accurate MRI diagnosis. This study aimed to evaluate whether 3.0T brain MRI with compressed sensing deep learning reconstruction (CS‑DLR) can reduce scanning time while maintaining diagnostic image quality.

**Methods:**

We retrospectively enrolled 69 patients with acute ischemic stroke who underwent 3.0T MRI. Conventional images and CS‑DLR reconstructed images at three acceleration rates (R3, R4, R5) were compared for AX T2WI, AX T1WI, and AX T2W FLAIR sequences. Two radiologists performed qualitative evaluation (5‑point scale: overall quality, noise, clarity). Quantitative assessment included signal‑to‑noise ratio (SNR) and contrast‑to‑noise ratio (CNR). Statistical analysis was performed using generalized estimating equation (GEE).

**Results:**

CS‑DLR significantly reduced scanning time. Quantitative analysis showed that SNR and CNR were significantly higher in the CS‑DLR groups than in the conventional group (*P* < 0.05). Qualitative scores for overall quality, noise, and clarity were significantly better in CS‑DLR R3 and R4 than in the conventional group (*P* < 0.05). CS‑DLR R5 showed similar overall image quality (*P* > 0.05) but significantly lower clarity (*P* < 0.05).

**Conclusion:**

CS‑DLR markedly shortens 3.0T MRI scanning time for acute ischemic stroke. CS‑DLR R3 and R4 provide superior image quality compared with conventional MRI, whereas R5 achieves comparable overall quality. CS‑DLR is clinically feasible and valuable for rapid emergency neuroimaging.

## Introduction

Acute ischemic stroke is the most common type of stroke. As a common emergency in neurology, it is characterized by high incidence, high disability rate and high mortality rate. Acute ischemic stroke has a sudden onset. After the brain tissue is ischemic and hypoxic, irreversible damage occurs rapidly. Every minute of delayed treatment, about 1.9 million neurons die. Therefore, timely and accurate diagnosis is crucial to improve the prognosis of patients. Magnetic resonance imaging (MRI) plays a vital role in the diagnosis of ischemic stroke, especially the application of diffusion-weighted imaging (DWI) technology, which makes the detection of early ischemic lesions possible. DWI has extremely high sensitivity and specificity in the early detection of ischemic stroke. Studies have shown that DWI combined with perfusion-weighted imaging (PWI) can achieve a sensitivity of 97.5% for the diagnosis of acute ischemic stroke [1]. This high sensitivity makes DWI the preferred tool for diagnosing acute stroke. In the clinical management of acute ischemic stroke, ‘time is brain’. While 3.0T MRI has improved imaging speed, the long scan time of DWI remains a concern for treatment delays. Additionally, the high field strength improves SNR and CNR but may perform poorly in DWI contrast [2]. Although advanced techniques like high b-value or synthetic b-value DWI improve lesion conspicuity [3, 4], they often require additional scanning time, necessitating a difficult trade-off in emergency settings [5]. Crucially, the emergency diagnosis of stroke requires not only the detection of ischemia via DWI but also the rapid exclusion of ‘stroke mimics’, such as tumors, encephalitis, or metabolic disorders, which necessitate morphological sequences like T1WI and T2WI [6]. In current hyperacute protocols, these sequences are often omitted or abbreviated to save time. Therefore, utilizing CS-DLR to achieve ultrafast, high-quality acquisition of T1 and T2 images is vital to provide a comprehensive differential diagnosis without compromising the urgent revascularization window.

The introduction of deep learning reconstruction (DLR) technology provides new possibilities for solving these problems. By enhancing denoising, improving spatial resolution, and increasing efficiency, DLR technology has shown its superiority in multiple studies. DLR technology has shown great promise in accelerating MRI scan times. In one study, DLR combined with conventional acceleration techniques significantly improved the image quality of 3T brain MRI and shortened the scanning time [7]. DLR technology has been shown to improve image quality in accelerated and high-resolution imaging settings without compromising diagnostic performance [8].

Recent evidence suggests that DLR technology excels in improving image sharpness and lesion conspicuity across various neuroimaging protocols. As neuroimaging applications increasingly integrate functional and structural data to optimize clinical decision-making [9], the prioritization of denoising structural details allows DLR to provide a more reliable foundation for the rapid assessment of acute neurological changes than traditional iterative methods [10].

With the continuous deepening of DLR technology research, more and more studies focus on the use of DLR technology based on highly undersampled k-space data to achieve faster magnetic resonance imaging [11, 12]. For example, the Deep-ER technology embeds physical models through deep learning to achieve rapid high-resolution brain metabolic imaging. Its reconstruction speed is 600 times faster than traditional methods, while improving the spatial-spectral quality and the accuracy of metabolite quantification [13]. In terms of clinical application, DLR technology has also shown its potential. In the study of accelerated brain MRI, the DLR method not only shortened the scanning time, but also improved the image quality and the clarity of structural details [14]. Besides, studies on pediatric body MRI have shown that deep learning-based denoising image reconstruction technology can significantly improve the overall quality of images while shortening the scanning time [15]. These studies demonstrate that DLR technology has broad application prospects in improving the speed and quality of MRI imaging. In this study, a new compressed sensing DLR (CS-DLR) method was used to evaluate the effectiveness of CS-DLR in reducing MRI scanning time and improving image quality in patients with acute ischemic stroke by qualitative and quantitative comparison with conventional full-sampling MRI. The aim was to evaluate the application value of CS-DLR technology in MRI of acute ischemic stroke.

## Materials and methods

### Ethics statement

This retrospective study was conducted in accordance with the Declaration of Helsinki. Ethical approval was obtained from the local ethics committee (Approval No.2024kt–1w008). Written informed consent was waived due to the retrospective nature of this study.

### General materials

A retrospective analysis was performed on 180 patients who underwent 3.0T MRI (Vantage Titan, Canon Medical Systems, Japan) examination. In our institution, the official stroke code program follows a standardized protocol: multimodal CT (NCCT + CTA + CTP) is the primary triage tool for hyperacute stroke, while MRI, including DWI and MRA, is prioritized for patients with “wake-up stroke” or when stroke mimics are highly suspected. After screening, 69 patients were finally included (average age 66 ± 13 years, 30 females). Based on clinical and radiological findings, 42 patients (60.9%) had small lacunar infarcts, while 27 patients (39.1%) presented with medium or large territory infarcts. Furthermore, large vessel occlusion (LVO) was identified in 15 patients (21.7%) within the study cohort. Inclusion criteria: (1) complete clinical data; (2) clinical signs and imaging findings confirmed as acute ischemic infarctions by diffusion-weighted imaging (DWI); (3) informed consent to the examination items. Exclusion criteria: (1) the image quality was obviously poor with obvious artifacts; (2) the clinical signs of suspected acute cerebral infarction on imaging did not meet the criteria.

### Methods

#### MRI and CS-DLR method

The unfolded optimized neural network architecture is selected to train the CS-DLR model [10]. U-Net has better performance in image processing and image restoration tasks, so U-Net is used instead of CNN. The k-space was undersampled using a variable-density Poisson-disk random sampling pattern. The model was trained using 16,098 FSE2D slices (Vantage Elan/Vantage Galan3T/Vantage Titan3T, Canon Medical Systems, Otawara, Japan). In order to enhance the generalization ability of the model, the training queue contains different contrast data such as T2WI, FLAIR, T1WI, PDWI, and WFS of different anatomical structures. The AX T2WI, AX T1WI, and AX T2W FLAIR full-sampling multi-parameter brain data of each patient were obtained as the conventional group. All images were retrospectively undersampled and reconstructed by the CS-DLR network model (Fig. 1). The undersampling rates were 1/3 (CS-DLR R3), 1/4 (CS-DLR R4) and 1/5 (CS-DLR R5), respectively.

**Fig. 1:**
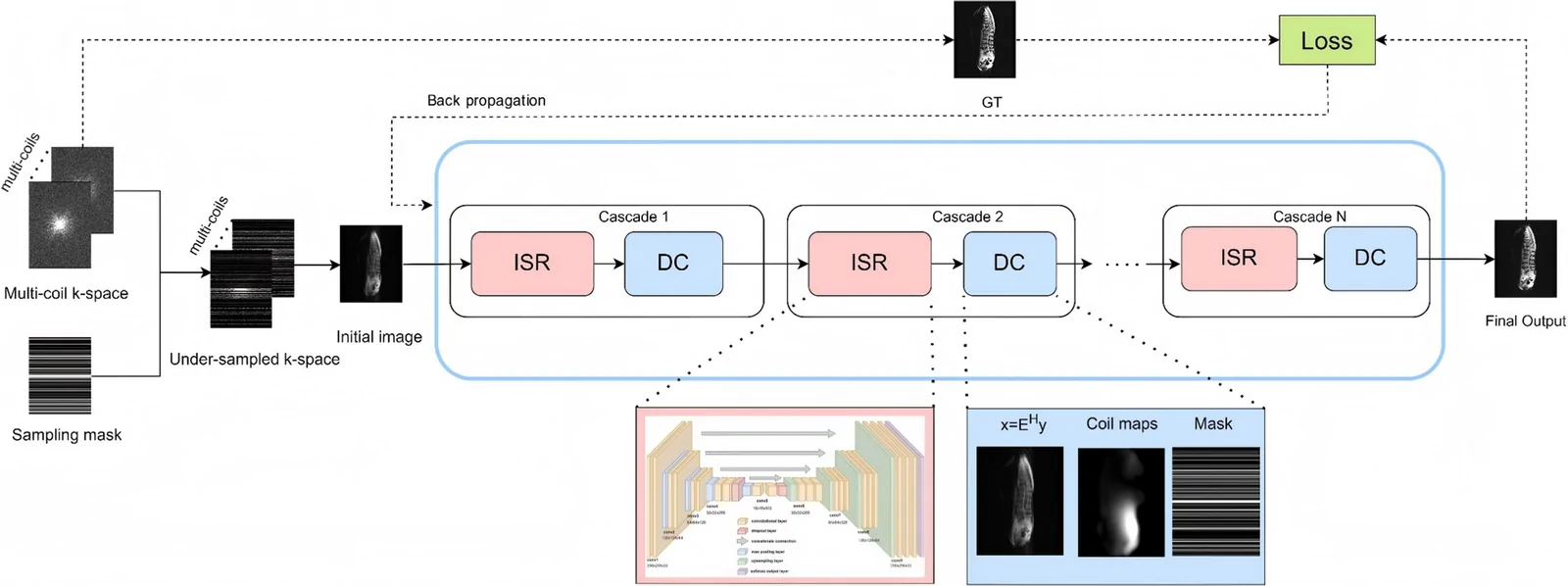
CS-DLR network model reconstruction and networking

ISR is the image space regularization neural network, and DC is the data consistency module. The entire reconstruction network consists of multiple cascaded sub-reconstruction networks. Each sub-reconstruction network contains a neural network and a data consistency module. The neural network updates the image in the image space, aiming to improve the image quality. At the same time, the data consistency module regularizes and optimizes the updated image to ensure a high degree of consistency with the original k-space sampling signal. After all the sub-reconstruction networks are processed, the final image is generated and output.

#### Image analysis

Two radiologists with five years of experience scored the overall image quality, noise, and clarity of the images in the routine group and the CS-DLR R3, CS-DLR R4, and CS-DLR R5 groups on a 5-point scale as a qualitative evaluation. In case of disagreement, the radiologists discussed and reached a consensus. (2) A senior radiologist with 10 years of experience and a radiologist with 5 years of experience delineated the regions of interest (ROIs) in the lesion area of the obtained images. The SNR and CNR between the lesion ROIs and the surrounding brain tissue of the conventional group and the CS-DLR group were calculated as quantitative indicators of the image.

### Statistical methods

SPSS 23.0 software was used for statistical analysis. Measurement data were described by mean ± sd. Generalized estimating equation (GEE) was used to evaluate the differences in qualitative and quantitative evaluation indices between the conventional and CS-DLR groups. To assess inter-observer reliability for qualitative scores, the intraclass correlation coefficient (ICC) was calculated. ICC values < 0.40, 0.40–0.75, and > 0.75 were considered to represent poor, fair-to-good, and excellent agreement, respectively. Furthermore, clinical diagnostic performance—including sensitivity, specificity, and negative predictive value (NPV)—was calculated for T1/T2-weighted images in identifying stroke mimics, using final clinical discharge diagnosis as the gold standard. The inspection level was α = 0.05.

## Results

### Quantitative scores of the conventional group and the CS-DLR group

Compared with the full k-space sampling, CS-DLR significantly shortened the acquisition time: AX T2W: 63s→21s/16/13s; AX T1W: 118s→39s/29s/24s; AX T2W FLAIR: 189s→63s/47s/38s; overall scanning time: 370s→123s/92s/75s. Overall, the SNR and CNR quality scores of CS-DLR were significantly better than those of the conventional group (*P* < 0.05; Table 1; Fig. 2).

**Table 1:** Comparison of SNR and CNR between the conventional group and the CS-DLR group (mean ± standard deviation)

Sequence types	Sampling mode	SNR	CNR
AX T_2_WI	Conventional group	74.01 ± 4.01	19.09 ± 1.91
	CS-DLR R3	119.18 ± 6.41^###^	29.70 ± 2.81^##^
	CS-DLR R4	141.49 ± 8.41^###^	34.89 ± 3.47^###^
	CS-DLR R5	167.02 ± 10.47^###^	38.01 ± 3.90^###^
	χ^2*^	114.121	30.795
	*P*	<0.001	<0.001
AX T1W	Conventional group	42.59 ± 2.52	9.40 ± 1.01
	CS-DLR R3	67.30 ± 3.89^###^	14.50 ± 1.60^##^
	CS-DLR R4	75.73 ± 4.24^###^	15.18 ± 1.65^##^
	CS-DLR R5	81.90 ± 4.60^###^	15.43 ± 2.15^##^
	χ^2*^	87.315	15.574
	*P*	<0.001	<0.05
AX T2W FLAIR	Conventional group	62.01 ± 3.41	19.34 ± 1.55
	CS-DLR R3	96.75 ± 4.44^###^	28.07 ± 2.11^###^
	CS-DLR R4	116.16 ± 5.58^###^	32.89 ± 2.50^###^
	CS-DLR R5	116.87 ± 5.55^###^	33.55 ± 2.70^###^
	χ^2*^	114.787	60.048
	*P*	<0.001	<0.001

*With the conventional group as the reference, the GEE was used to calculate χ^2^ to explore the relationship between different acceleration times. ##*P* < 0.05, ###*P* < 0.001; Conventional group: the image was acquired with full k-space sampling; CS DLR R3/R4/R5: the undersampling factors of the images were 1/3 (R3), 1/4 (R4) or 1/5 (R5); the images were reconstructed using the CS-DLR network

**Fig. 2:**
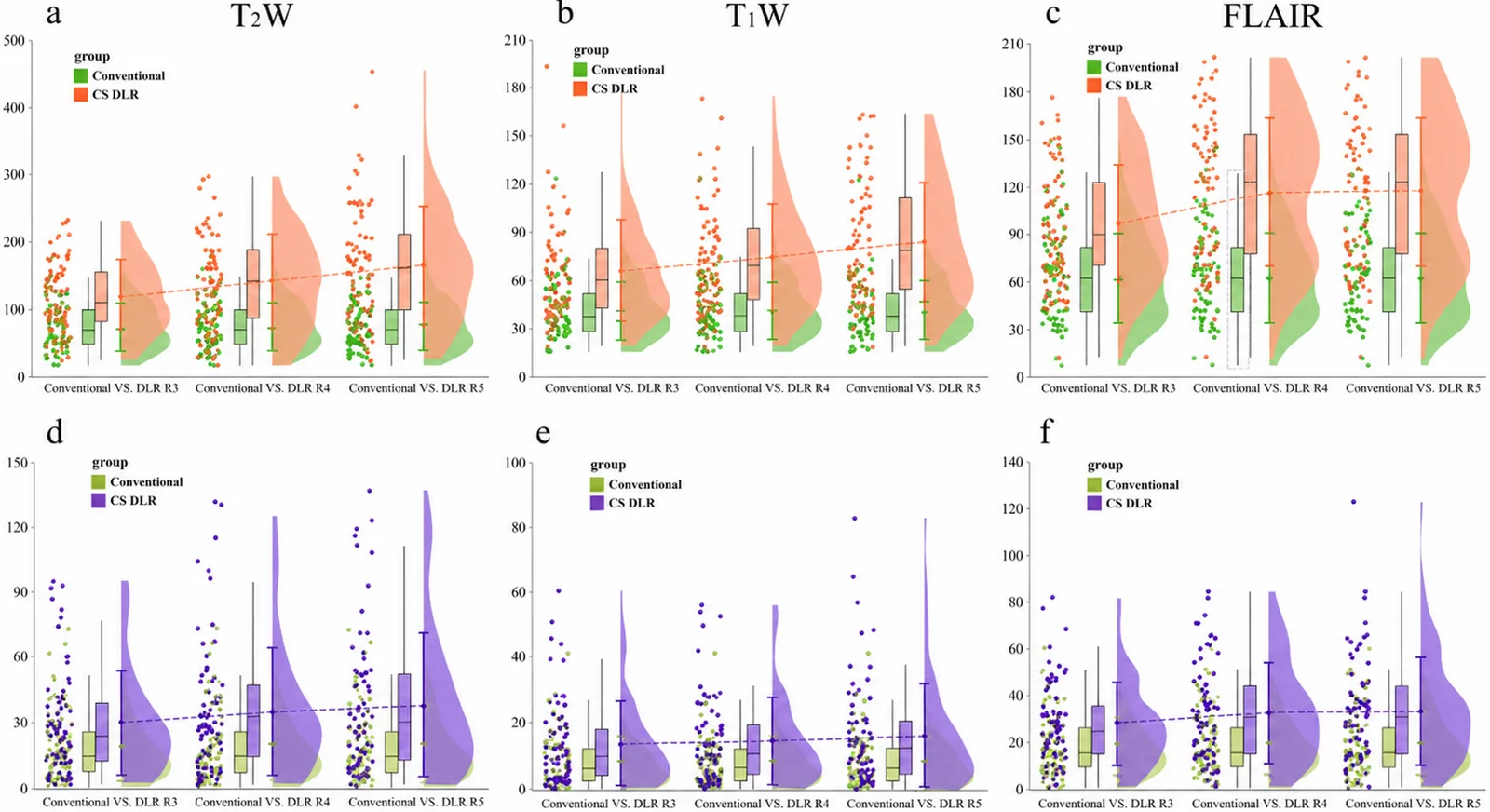
Quantitative analysis results of CS-DLR SNR and CNR quality scores. In the conventional group, the images were acquired with full k-space sampling; in the DLR R3/R4/R5 groups, the images were acquired with undersampling factors of 1/3 (R3), 1/4 (R4), or 1/5, and reconstructed using the CS-DLR network. SNR results (**a**-**c**); CNR results (**d**-**f**). SNR and CNR of T2WI (**a**, **d**); SNR and CNR of T1W sequence (**b**, **e**); SNR and CNR of T2W FLAIR sequence (**c**, **f**). The dashed line represents the mean

### Qualitative scoring results of the conventional group and the CS-DLR group

When CS-DLR R3 and R4 were applied, the overall image quality, noise, and clarity scores of the AX T2WI, AX T1WI, and AX T2W FLAIR full-sampling multi-parameter brain data of each patient were obtained as the conventional group. These conventional morphological sequences were specifically selected to evaluate the reconstruction performance and image quality of the CS-DLR model across a diverse range of tissue contrast mechanisms, and the differences were statistically significant (*P* < 0.05; Table 2; Fig. 3). When CS-DLR R5 was applied, there was no significant difference in the overall image quality score compared with the conventional group (*P* > 0.05). In terms of lesion display, there was no significant difference in clarity between CS-DLR R3 and CS-DLR R4 compared with the conventional group (*P* > 0.05). The clarity of CS-DLR R5 was significantly lower than that of the conventional group (*P* < 0.05).

**Table 2:** Qualitative scores of MRI images in the conventional group and the CS-DLR group (mean ± standard deviation)

Sequence types	Sampling mode	Overall image quality	Noise	Clarity
AX T_2_WI	Conventional group	3.96 ± 0.11	3.93 ± 0.11	4.30 ± 0.08
	DLR R3	4.48 ± 0.08^###^	4.45 ± 0.08^###^	4.35 ± 0.08
	DLR R4	4.45 ± 0.09^###^	4.42 ± 0.09^###^	4.28 ± 0.09
	DLR R5	3.99 ± 0.10	4.59 ± 0.05^###^	3.65 ± 0.08^###^
	χ^2*^	27.367	29.746	47.798
	*P*	<0.001	<0.001	<0.001
AX T1W	Conventional group	3.93 ± 0.11	3.88 ± 0.11	4.19 ± 0.07
	DLR R3	4.30 ± 0.08^##^	4.23 ± 0.77^##^	4.16 ± 0.07
	DLR R4	4.28 ± 0.08^##^	4.28 ± 0.08^##^	4.04 ± 0.08
	DLR R5	3.87 ± 0.09	4.65 ± 0.06^###^	3.52 ± 0.07^###^
	χ^2*^	19.766	50.025	55.509
	*P*	<0.001	<0.001	<0.001
AX T_2_WI FLAIR	Conventional group	3.93 ± 0.11	3.70 ± 0.10	4.25 ± 0.08
	DLR R3	4.45 ± 0.08^###^	4.25 ± 0.08^###^	4.10 ± 0.07
	DLR R4	4.33 ± 0.09^##^	4.22 ± 0.08^###^	3.90 ± 0.08^##^
	DLR R5	3.90 ± 0.10	4.57 ± 0.06^###^	3.54 ± 0.07^###^
	χ^2*^	27.371	60.048	52.173
	*P*	<0.001	<0.001	<0.001

*Using the conventional group as a reference, the GEE was used to calculate χ^2^ to explore the relationship between different acceleration times. ##*P* < 0.05, ###*P* < 0.001; Conventional group: the image was acquired with full k-space sampling; CS DLR R3/R4/R5: the undersampling factors of the images were 1/3 (R3), 1/4 (R4) or 1/5 (R5); the images were reconstructed using the CS-DLR network

**Fig. 3:**
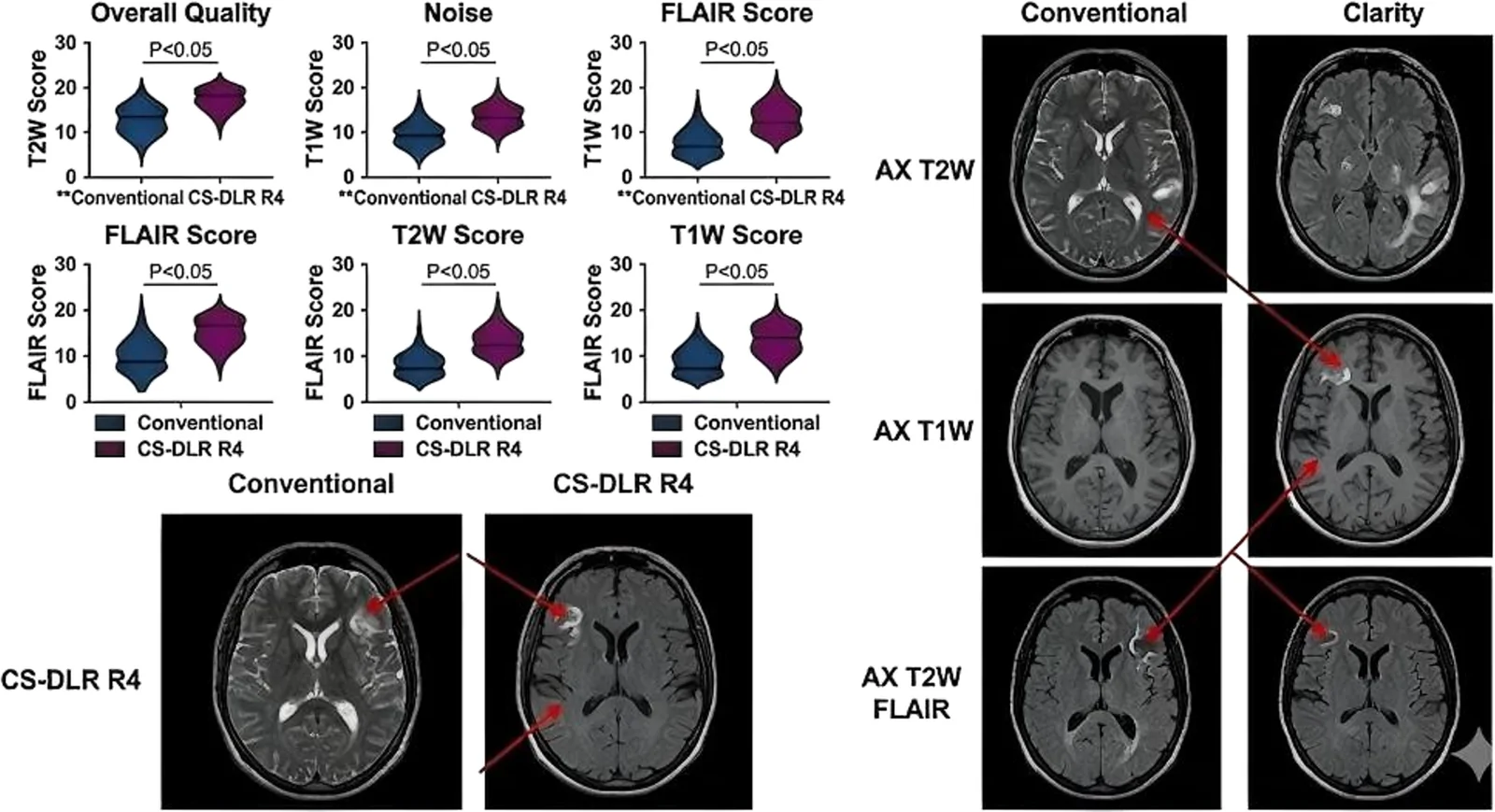
Qualitative scoring results of the conventional group and the CS-DLR group. In the conventional group, the images were acquired with full k-space sampling; in the DLR R3/R4/R5 groups, the images were acquired with undersampling factors of 1/3 (R3), 1/4 (R4) or 1/5 (R5), and reconstructed using the CS-DLR network. The dashed lines indicate the mean

### Inter-observer agreement and diagnostic performance

The inter-observer agreement for qualitative image quality scores (overall quality, noise, and clarity) was excellent, with ICC values ranging from 0.81 to 0.88 across all sequences. Specifically, the ICC for AX T2WI and FLAIR clarity reached 0.86 and 0.84, respectively. Regarding clinical utility, the diagnostic performance of the optimized CS-DLR T1/T2 protocol for excluding stroke mimics showed a sensitivity of 92.8%, a specificity of 94.2%, and an NPV of 95.1%. There was no statistically significant difference in diagnostic accuracy between the CS-DLR R4 group and the conventional group (*P* > 0.05).

## Discussion

Acute ischemic stroke (AIS) is one of the leading causes of death and disability worldwide, and the timeliness of its diagnosis and treatment directly affects the prognosis of patients [6]. In the diagnosis of acute ischemic stroke, it is crucial to quickly distinguish true stroke from non-vascular diseases with similar symptoms (i.e., “stroke mimics”) because the effectiveness of treatment is closely related to time. MRI plays a vital role in the diagnosis of ischemic stroke, especially with the application of DWI technology, which makes it possible to detect early ischemic lesions. However, the time required for conventional 3.0T MRI parameters plus DWI scanning remains long in emergency settings, posing a challenge to rapid diagnosis. The clinical significance of shortening T1 and T2 duration lies in the rapid exclusion of “stroke mimics.” In hyperacute scenarios, clinicians must quickly differentiate true ischemia from mimics to avoid inappropriate thrombolysis. Focusing on T1, T2, and FLAIR sequences in this initial stage was a strategic choice to verify the CS-DLR algorithm’s robustness across various fundamental tissue contrasts. While DWI is indispensable for ischemia detection, these conventional morphological sequences often represent a significant time bottleneck in standard stroke protocols. By compressing T1 and T2 acquisition times into seconds, CS-DLR allows for a comprehensive diagnostic protocol that traditionally would have been too time-consuming for emergency use. The results of this study showed that the CS-DLR technology significantly shortened the scanning time on 3.0T MRI while maintaining good image quality. This finding is consistent with the results of several studies, which found that deep learning-accelerated MRI can significantly reduce examination time and show diagnostic performance comparable to traditional MRI in acute ischemic stroke [16]. In addition, image reconstruction methods based on compressed sensing (CS) or deep learning (DL)are superior to traditional methods in performance [17]. The results of this work further validated these findings, especially when the image quality (including overall quality, noise, and clarity) of the 3-fold (R3) and 4-fold (R4) reconstructions was significantly better than that of conventional MRI, which is consistent with the advantages of DLR technology in resolution and artifact reduction [18].

The advantages of CS-DLR technology may stem from its combination of the dual advantages of CS and DL. CS reduces the amount of data acquisition through sparse sampling, while deep learning reconstructs high-quality images from sparse data by training neural networks. Studies have found that deep learning technology performs better than traditional CS-based regularization methods in accelerating MRI reconstruction, especially in parameter tuning [19]. The CS-DLR technique in this study may maintain the high quality of the images while reducing the scanning time through a similar mechanism. In addition, self-supervised deep learning methods have performed well in MRI reconstruction, and can generate high-quality parameter maps close to the reference images, which is consistent with the excellent performance of CS-DLR R3 and R4 in this study [20].

In this study, CS-DLR technology was applied to 3.0T MRI scanning of acute ischemic stroke, and the influence of different acceleration factors (R3, R4, R5) on image quality was systematically evaluated. This study provides a feasible technical solution for the rapid diagnosis of acute ischemic stroke. Regarding the reviewer’s query on MRA, although standardized MRA data were not available for all 69 patients in this specific retrospective cohort, we recognize its critical role in identifying large vessel occlusion. Theoretically, the CS-DLR technology demonstrated in this study is highly applicable to MRA sequences. By utilizing similar deep learning-based undersampling reconstruction, the acquisition of high-resolution 3D MRA could be significantly accelerated, further enhancing the efficiency of the MRI-based stroke code program. Shortening the scan time speeds up diagnosis and treatment decisions in emergency situations. While the performance of DL reconstruction has been demonstrated across diverse clinical domains [21], the high-quality image data generated by CS-DLR provides a necessary foundation for the further development of deep learning models in acute stroke care [22]. Furthermore, DLR algorithms effectively address the essential clinical demand for accelerated imaging protocols, and their optimized application within acute neuroimaging remains a priority to bridge existing research gaps in emergency stroke protocols [23]. This work fills this gap and provides a new technical solution for rapid MRI scanning of acute ischemic stroke. Furthermore, previous studies have highlighted the potential of DLR technology in real-time clinical-grade MRI reconstruction [24], our study validates this potential through actual clinical data in the setting of acute ischemic stroke.

Despite the positive results of these findings, there are still some limitations. First, the sample size was relatively small (69 cases). This limited sample size precluded a robust subgroup analysis to evaluate how variables such as infarct size or the presence of large vessel occlusion (LVO) might influence the performance of CS-DLR, which may affect the universality of the results and limit the overall statistical power. Second, this study was a single-center retrospective study exclusively utilizing a 3.0T MRI scanner; therefore, the generalizability of these findings to other field-strength systems, such as 1.5T MRI, remains to be validated in future multi-center studies. In addition, the DLR method is highly dependent on the training data, which may not cover all possible pathological changes, potentially affecting the model’s stability. Third, this study only evaluated conventional morphological sequences and did not include DWI or SWI, which are core components of acute stroke protocols. This preliminary verification was a strategic choice to test the model on diverse contrasts, but we plan to extend this technology to functional sequences in subsequent research.

## Conclusions

The CS-DLR technique significantly shortens the scanning time. Compared with conventional MRI, CS-DLR R3 or R4 has better image quality, and CS-DLR R5 has comparable image quality. Acute ischemic stroke can be comprehensively evaluated in CS-DLR images, which can be applied in the judgment of acute ischemic stroke.

## Data Availability

The data that support the findings of this study are available from the corresponding author upon reasonable request.
